# Supplementary material for: SyNDI: synchronous network data integration framework
Source: BMC Bioinformatics. 2018 Nov 6;19:403. doi: 10.1186/s12859-018-2426-5 (PMC6219086; doi:10.1186/s12859-018-2426-5)
Supplement: Supplementary file 5 — Files of the example presented in Additional file 4. This additional file is a zip package that contains all files used in Additional file 4. Detailed descriptions of each of these files are presented in a README file included in this zip package. (GZ 30411 kb) [file 12859_2018_2426_MOESM5_ESM.gz › SyNDITools/Meme2Fimo/meme_4.9.0_4.tar.gz!/meme_4.9.0/tests/common/crp0.meme.html]

MEME


---

|  |  |  |  |  |  |
| --- | --- | --- | --- | --- | --- |
| **Command line** | **Training Set** | **First Motif** | **Summary of Motifs** | **Termination** | **Explanation** |

  
 **Search sequence databases for the best combined matches with these motifs using
MAST.  
Search sequence databases for all matches with these motifs using
FIMO.
  
Find Genome Ontology terms associated
with upstream sequences matching these motifs using
GOMO.
  
Submit these motifs to
BLOCKS multiple alignment processor.**   

---


**MEME - Motif discovery tool**


---

MEME version 4.2.0 (Release date: Thu Apr 9 11:15:56 PDT 2009)

For further information on how to interpret these results or to get
a copy of the MEME software please access
http://meme.sdsc.edu.

This file may be used as input to the MAST algorithm for searching
sequence databases for matches to groups of motifs. MAST is available
for interactive use and downloading at
http://meme.sdsc.edu.

---


**REFERENCE**


---

If you use this program in your research, please cite:

#### Timothy L. Bailey and Charles Elkan, "Fitting a mixture model by expectation maximization to discover motifs in biopolymers", Proceedings of the Second International Conference on Intelligent Systems for Molecular Biology, pp. 28-36, AAAI Press, Menlo Park, California, 1994.

---


---

**TRAINING SET**


---

|  |  |  |  |  |  |
| --- | --- | --- | --- | --- | --- |
| DATAFILE= tests/common/crp0.fasta |  |  |  |  |  |
| ALPHABET= ACGT |  |  |  |  |  |
| Sequence name | Weight | Length | Sequence name | Weight | Length |
| ce1cg | 1.0000 | 105 | ara | 1.0000 | 105 |
| bglr1 | 1.0000 | 105 | crp | 1.0000 | 105 |
| cya | 1.0000 | 105 | deop2 | 1.0000 | 105 |
| gale | 1.0000 | 105 | ilv | 1.0000 | 105 |
| lac | 1.0000 | 105 | male | 1.0000 | 105 |
| malk | 1.0000 | 105 | malt | 1.0000 | 105 |
| ompa | 1.0000 | 105 | tnaa | 1.0000 | 105 |
| uxu1 | 1.0000 | 105 | pbr322 | 1.0000 | 105 |
| trn9cat | 1.0000 | 105 | tdc | 1.0000 | 105 |


---


**COMMAND LINE SUMMARY**


---

```
This information can also be useful in the event you wish to report a
problem with the MEME software.

command: meme tests/common/crp0.fasta -dna -nmotifs 3 -revcomp
```

|  |  |  |  |  |  |  |  |  |  |
| --- | --- | --- | --- | --- | --- | --- | --- | --- | --- |
| model: | mod= | zoops | nmotifs= | 3 | evt= | inf |  |  |  |
| object function= E-value of product of p-values | | | |  |  |  |  |  |  |
| width: | minw= | 8 | maxw= | 50 | minic= | 0.00 |  |  |  |
| width: | wg= | 11 | ws= | 1 | endgaps= | yes |  |  |  |
| nsites: | minsites= | 2 | maxsites= | 18 | wnsites= | 0.8 |  |  |  |
| theta: | prob= | 1 | spmap= | uni | spfuzz= | 0.5 |  |  |  |
| em: | prior= | dirichlet | b= | 0.01 | maxiter= | 50 |  |  |  |
|  | distance= | 1e-05 |  |  |  |  |  |  |  |
| data: | n= | 1890 | N= | 18 |  |  |  |  |  |
| strands: + - |  |  |  |  |  |  |  |  |  |
| sample: | seed= | 0 | seqfrac= | 1 |  |  |  |  |  |
| Letter frequencies in dataset: | | | |  |  |  |  |  |  |
| A 0.304 C 0.196 G 0.196 T 0.304 | | | |  |  |  |  |  |  |
| Background letter frequencies (from dataset with add-one prior applied):  A 0.304 C 0.196 G 0.196 T 0.304 | | | |  |  |  |  |  |  |

---


|  |  |
| --- | --- |
| **P** | **N** |

**MOTIF 1
width = 18
sites = 18
llr = 180
E-value = 1.1e-006** 


---

SEQUENCE LOGO

Information Content

**14.7 (bits)**

Relative Entropy

**14.4 (bits)**

Download LOGO

**Without SSC:**[EPS][PNG]

**With SSC:**[EPS][PNG]

| NAME | STRAND | START | P-VALUE | SITES |  |  |
| --- | --- | --- | --- | --- | --- | --- |
| ara | - | 58 | 2.51e-07 | TGGCATAGCA | **AAGTGTGACGCCGTGCAA** | ATAATCAATG |
| lac | + | 8 | 5.35e-07 | AACGCAAT | **TAATGTGAGTTAGCTCAC** | TCATTAGGCA |
| malt | + | 40 | 8.61e-07 | AAAGATTTGG | **AATTGTGACACAGTGCAA** | ATTCAGACAC |
| ilv | - | 42 | 1.69e-06 | GCAAAGGGAA | **AATTGAGGGGTTGATCAC** | GTTTTGTACT |
| pbr322 | - | 56 | 2.85e-06 | CTCCTTACGC | **ATCTGTGCGGTATTTCAC** | ACCGCATATG |
| deop2 | + | 59 | 2.85e-06 | AGATTTCCTT | **AATTGTGATGTGTATCGA** | AGTGTGTTGC |
| uxu1 | + | 16 | 5.17e-06 | AGAGTGAAAT | **TGTTGTGATGTGGTTAAC** | CCAATTAGAA |
| trn9cat | + | 83 | 5.69e-06 | CTTTTGGCGA | **AAATGAGACGTTGATCGG** | CACG |
| ce1cg | - | 64 | 7.54e-06 | GGACTTCCAT | **TTTTGTGAAAACGATCAA** | AAAAACAGTC |
| ompa | + | 47 | 9.04e-06 | TTTTTTTCAT | **ATGCCTGACGGAGTTCAC** | ACTTGTAAGT |
| crp | - | 66 | 9.89e-06 | TACTGCACGG | **TAATGTGACGTCCTTTGC** | ATACATGCAG |
| male | + | 13 | 1.29e-05 | TTACCGCCAA | **TTCTGTAACAGAGATCAC** | ACAAAGCGAC |
| gale | - | 45 | 1.41e-05 | AAGATGCGAA | **AAGTGTGACATGGAATAA** | ATTAGTGGAA |
| tdc | - | 81 | 1.53e-05 | AACAGG | **ATATGTGCGACCACTCAC** | AAATTAACTT |
| malk | + | 60 | 1.67e-05 | ATGTAAGGAA | **TTTCGTGATGTTGCTTGC** | AAAAATCGTG |
| cya | + | 49 | 1.81e-05 | TCAATCAGCA | **AGGTGTTAAATTGATCAC** | GTTTTAGACC |
| tnaa | + | 70 | 2.73e-05 | CTCCCCGAAC | **GATTGTGATTCGATTCAC** | ATTTAAACAA |
| bglr1 | + | 75 | 8.30e-05 | CAAAGTTAAT | **AACTGTGAGCATGGTCAT** | ATTTTTATCA |

---

**Motif 1 block diagrams**


---

| Name | Lowest p-value | Motifs |
| --- | --- | --- |
| ara | 2.51e-07 | --- \| -1 \| --- |
| lac | 5.35e-07 | --- \| +1 \| --- |
| malt | 8.61e-07 | --- \| +1 \| --- |
| ilv | 1.69e-06 | --- \| -1 \| --- |
| pbr322 | 2.85e-06 | --- \| -1 \| --- |
| deop2 | 2.85e-06 | --- \| +1 \| --- |
| uxu1 | 5.17e-06 | --- \| +1 \| --- |
| trn9cat | 5.69e-06 | --- \| +1 \| --- |
| ce1cg | 7.54e-06 | --- \| -1 \| --- |
| ompa | 9.04e-06 | --- \| +1 \| --- |
| crp | 9.89e-06 | --- \| -1 \| --- |
| male | 1.29e-05 | --- \| +1 \| --- |
| gale | 1.41e-05 | --- \| -1 \| --- |
| tdc | 1.53e-05 | --- \| -1 \| --- |
| malk | 1.67e-05 | --- \| +1 \| --- |
| cya | 1.81e-05 | --- \| +1 \| --- |
| tnaa | 2.73e-05 | --- \| +1 \| --- |
| bglr1 | 8.30e-05 | --- \| +1 \| --- |
| SCALE | | \| \| \| \| \| \| \| \| \|; 1 \| 25 \| 50 \| 75 \| 100 |

---

**Motif 1 in BLOCKS format**


---


  
 **to BLOCKS multiple alignment processor.**


---


**Motif 1 position-specific scoring matrix**


---


  
 **Scan sequence databases for the best match in each sequence using MAST.**


---


**Motif 1 position-specific probability matrix** 


---


  
 **Scan sequence databases for all matches with this motif using FIMO.**  
 **Compare to known motifs in motif databases using
Tomtom.**  
 **Find Genome Ontology terms associated with
upstream regions matching this motif using
GOMO.**

---

**Motif 1 regular-expression**


---

 **[AT][AT][TAG]TGTGA[CGT][GA][TC][ATCGC]G[AT]TC[AG][CA]**   

---


Time 1.75 secs.
  

---


|  |  |
| --- | --- |
| **P** | **N** |

**MOTIF 2
width = 8
sites = 2
llr = 24
E-value = 1.5e+004** 


---

SEQUENCE LOGO

Information Content

**15.0 (bits)**

Relative Entropy

**17.5 (bits)**

Download LOGO

**Without SSC:**[EPS][PNG]

**With SSC:**[EPS][PNG]

| NAME | STRAND | START | P-VALUE | SITES |  |  |
| --- | --- | --- | --- | --- | --- | --- |
| ilv | + | 4 | 2.18e-06 | GCTC | **CGGCGGGG** | TTTTTTGTTA |
| male | + | 40 | 5.56e-06 | CACAAAGCGA | **CGGTGGGG** | CGTAGGGGCA |

---

**Motif 2 block diagrams**


---

| Name | Lowest p-value | Motifs |
| --- | --- | --- |
| ilv | 2.18e-06 | --- \| +2 \| --- |
| male | 5.56e-06 | --- \| +2 \| --- |
| SCALE | | \| \| \| \| \| \| \| \| \|; 1 \| 25 \| 50 \| 75 \| 100 |

---

**Motif 2 in BLOCKS format**


---


  
 **to BLOCKS multiple alignment processor.**


---


**Motif 2 position-specific scoring matrix**


---


  
 **Scan sequence databases for the best match in each sequence using MAST.**


---


**Motif 2 position-specific probability matrix** 


---


  
 **Scan sequence databases for all matches with this motif using FIMO.**  
 **Compare to known motifs in motif databases using
Tomtom.**  
 **Find Genome Ontology terms associated with
upstream regions matching this motif using
GOMO.**

---

**Motif 2 regular-expression**


---

 **CGG[CT]GGGG**   

---


Time 2.94 secs.
  

---


|  |  |
| --- | --- |
| **P** | **N** |

**MOTIF 3
width = 11
sites = 2
llr = 31
E-value = 2.0e+004** 


---

SEQUENCE LOGO

Information Content

**20.0 (bits)**

Relative Entropy

**22.3 (bits)**

Download LOGO

**Without SSC:**[EPS][PNG]

**With SSC:**[EPS][PNG]

| NAME | STRAND | START | P-VALUE | SITES |  |  |
| --- | --- | --- | --- | --- | --- | --- |
| lac | - | 32 | 7.89e-08 | AAGTGTAAAG | **CCTGGGGTGCC** | TAATGAGTGA |
| trn9cat | + | 35 | 2.01e-07 | ATAAATAAAT | **CCTGGTGTCCC** | TGTTGATACC |

---

**Motif 3 block diagrams**


---

| Name | Lowest p-value | Motifs |
| --- | --- | --- |
| lac | 7.89e-08 | --- \| -3 \| --- |
| trn9cat | 2.01e-07 | --- \| +3 \| --- |
| SCALE | | \| \| \| \| \| \| \| \| \|; 1 \| 25 \| 50 \| 75 \| 100 |

---

**Motif 3 in BLOCKS format**


---


  
 **to BLOCKS multiple alignment processor.**


---


**Motif 3 position-specific scoring matrix**


---


  
 **Scan sequence databases for the best match in each sequence using MAST.**


---


**Motif 3 position-specific probability matrix** 


---


  
 **Scan sequence databases for all matches with this motif using FIMO.**  
 **Compare to known motifs in motif databases using
Tomtom.**  
 **Find Genome Ontology terms associated with
upstream regions matching this motif using
GOMO.**

---

**Motif 3 regular-expression**


---

 **CCTGG[GT]GT[CG]CC**   

---


Time 4.08 secs.
  

---

|  |  |
| --- | --- |
| **P** | **N** |

**SUMMARY OF MOTIFS**


---


---

 **Combined block diagrams: non-overlapping sites with p-value <
0.0001**


---

| Name | Combined p-value | Motifs |  |  |  |
| --- | --- | --- | --- | --- | --- |
| ce1cg | 7.53e-03 | --- \| -1 \| --- | | | |
| ara | 9.02e-04 | --- \| -1 \| --- | | | |
| bglr1 | 2.01e-01 | --- \| +1 \| --- | | | |
| crp | 3.77e-02 | --- \| -1 \| --- | | | |
| cya | 6.49e-02 | --- \| +1 \| --- | | | |
| deop2 | 1.19e-02 | --- \| +1 \| --- | | | |
| gale | 6.15e-02 | --- \| -1 \| --- | | | |
| ilv | 4.50e-06 | --- \| +2 \| --- \| -1 \| --- | | | |
| lac | 1.93e-07 | --- \| +1 \| --- \| -3 \| --- | | | |
| male | 1.22e-04 | --- \| +1 \| --- \| +2 \| --- | | | |
| malk | 1.39e-02 | --- \| +1 \| --- | | | |
| malt | 5.01e-03 | --- \| +1 \| --- | | | |
| ompa | 3.94e-02 | --- \| +1 \| --- | | | |
| tnaa | 2.80e-02 | --- \| -1 \| --- | | | |
| uxu1 | 1.70e-02 | --- \| +1 \| --- | | | |
| pbr322 | 6.62e-03 | --- \| -1 \| --- | | | |
| trn9cat | 5.41e-06 | --- \| +3 \| --- \| +1 \| --- | | | |
| tdc | 6.53e-02 | --- \| -1 \| --- | | | |
| SCALE | | \| \| \| \| \| \| \| \| \|; 1 \| 25 \| 50 \| 75 \| 100 |  |  |  |

  
**Motif summary in machine readable format.**

---

**Stopped
because Stopped because nmotifs = 3 reached..** 


---

  
  
CPU: Macintosh.local  
  

---

### EXPLANATION OF MEME RESULTS

---

#### The MEME results consist of:

- The **version**
  of MEME and the date it was released.
- The **reference**
  to cite if you use MEME in your research.
- A description of the
  **sequences**
  you submitted (the "training set") showing the name,
  "weight" and length of each sequence.
- The **command line summary**
  detailing the parameters with which you ran MEME.
- Information on each of the **motifs**
  MEME discovered, including:
  1. A summary line
     showing the width, number of occurrences, log likelihood ratio
     and statistical significance of the motif.
  2. A  sequence LOGO.
  3. The  information content
     of the motif.
  4. The  relative entropy
     of the motif.
  5. Downloadable LOGO files
     suitable for publication.
  6. The
     occurrences of the motif
     sorted by *p*-value and aligned with each other.
  7. Block diagrams
     of the occurrences of the motif within each sequence in the
     training set.
  8. The motif in
     BLOCKS or FASTA format.
  9. A 
     position-specific scoring matrix (PSSM)
     for use by the MAST
     database search program.
  10. The 
      position specific probability matrix (PSPM)
      describing the motif.
  11. A 
      regular expression describing the motif.
- A **summary of motifs**
  showing an optimized (non-overlapping)
  tiling
  of all of the motifs onto each of the sequences in the training set.
- The reason why MEME stopped
  and the name of the CPU on which it ran.
- This **explanation** of how to interpret MEME results.

MOTIFS

For each motif that it discovers in the training set,
MEME prints the following information:

- #### Summary Line

  This line gives the width (**'width'**),
  number of occurrences in the training set (**'sites'**),
  log likelihood ratio (**'llr'**) and
  **'*E*-value'** of the motif.
  Each motif describes a pattern of a fixed width--no gaps are
  allowed in MEME motifs.
  MEME numbers the motifs consecutively from one as it finds them.
  MEME usually finds the most statistically significant
  (low *E*-value) motifs first.
  The statistical significance of a motif is based on its log
  likelihood ratio, its width and number of occurrences, the
  background letter frequencies (given in the
  command line summary
  ), and the size of the training set. The *E*-value is an
  estimate of the expected number of motifs with the given log
  likelihood ratio (or higher), and with the same width and number of
  occurrences, that one would find in a similarly sized set of random
  sequences.
  (In random sequences each position is independent with letters chosen
  according to the background letter frequencies.) The log likelihood
  ratio is the logarithm of the ratio of the probability of the
  occurrences of the motif given the motif model (likelihood given the
  motif) versus their probability given the background model
  (likelihood given the null model).
  (Normally the background model is a 0-order Markov model
  using the background letter frequencies, but higher order Markov
  models may be specified via the **-bfile** option to MEME.)
  Clicking on the **buttons** to the left of the motif summary line
  takes you to the previous motif (P) or next motif (N).
- #### Sequence LOGO

  MEME motifs are represented by position-specific probability matrices
  that specify the probability of each possible letter appearing at each
  possible position in an occurrence of the motif. These are displayed
  as "sequence LOGOS", containing stacks of letters at each position
  in the motif. The total height of the stack is the "information
  content" of that position in the motif in bits. The height of the
  individual letters in a stack is the probability of the letter at that
  position multiplied by the total information content of the stack.

  Note:
  The MEME LOGO differs from those produced by the
  Weblogo program
  because a **small-sample correction is NOT applied**.
  However, MEME LOGOs in PNG and encapsulated postscript (EPS) formats
  **with small-sample correction (SSC)** are available by clicking
  on one of the links named "With SSC" (EPS or PNG) under
  Download LOGO.
  The MEME LOGOs without small sample correction are similarly available.
  Error bars are included in the LOGOs with small-sample correction.

  The information content of each motif position is computed
  as described in the paper by Schneider and Stephens,
  "Sequence Logos: A New Way to Display Consensus Sequences" but
  **the small-sample correction, e(n),
  is set to zero for the LOGO displayed in the MEME output.**
  The corrected information content of position i is given by

  ```
             R(i) for amino acids   = log2(20) - (H(i) + e(n))   (1a) 
             R(i) for nucleic acids =    2    - (H(i) + e(n))    (1b)
  ```

  where H(i) is the entropy of position i,

  ```
             H(l) = - (Sum f(a,i) * log2[ f(a,i) ]).             (2)
  ```

  Here, f(a,i) is the frequency of base or amino acid a
  at position i, and e(n) is the
  small-sample correction for an alignment of n letters.
  The height of letter a in column i is given by

  ```
             height = f(a,i) * R(i)                              (3)
  ```

  The approximation for the small-sample correction, e(n),
  is given by:

  ```
             e(n) = (s-1) / (2 * ln(2) * n),                     (4)
  ```

  where s is 4 for nucleotides, 20 for amino acids, and
  n is the number of sequences in the alignment.

  The letters in the logos are colored as follows.
  For DNA sequences, the letter categories contain one letter
  each. For proteins, the categories are based on the biochemical properties
  of the various amino acids. The categories and their colors are:

  | NUCLEIC ACIDS | COLOR |
  | --- | --- |
  | A | RED |
  | C | BLUE |
  | G | ORANGE |
  | T | GREEN |

  | AMINO ACIDS | COLOR | PROPERTIES |
  | --- | --- | --- |
  | A, C, F, I, L, V, W and M | BLUE | Most hydrophobic[Kyte and Doolittle, 1982] |
  | NQST | GREEN | Polar, non-charged, non-aliphatic residues |
  | DE | MAGENTA | Acidic |
  | KR | RED | Positively charged |
  | H | PINK |  |
  | G | ORANGE |  |
  | P | YELLOW |  |
  | Y | TURQUOISE |  |

  J. Kyte and R. Doolittle, 1982.
  "A Simple Method for Displaying the Hydropathic Character of a Protein",
  J. Mol Biol. 157, 105-132.

  **Note:** the "text" output format of
  MEME preserves the historical MEME format where LOGOS
  are replaced by a simplified probability matrix,
  a relative entropy plot, and a multi-level
  consensus sequence.
- #### Information Content

  This is the information content of the motif in bits.
  It is equal to the sum of the **uncorrected**
  information content, R(),
  in the columns of the LOGO.
  This is equal relative entropy of the motif relative to a
  uniform background frequency model.
- #### Relative Entropy

  This is the relative entropy of the motif,
  computed in bits and relative to the background
  letter frequencies given in the
  command line summary. It is equal to
  the log-likelihood ratio (llr)
  divided by the number of
  occurrences (sites)
  of the motif times 1/ln(2),

  ```
                 re = llr / (sites * ln(2)).
  	
  ```
- #### Occurrences of the Motif

  MEME displays the occurrences (sites) of the motif in the
  training set.
  The sites are shown aligned with each other, and the ten sequence
  positions preceding and following each site are also shown.
  Each site is identified by the name of the sequence where it occurs,
  the strand (if both strands of DNA sequences are being used), and the
  position in the sequence where the site begins. When the DNA strand
  is specified, '+' means the sequence in the training set,
  and '-' means the reverse complement of the training set sequence.
  (For '-' strands, the 'start' position is actually the position on the
  **positive** strand where the site ends.)
  The sites are listed in order of increasing statistical significance
  (*p*-value). The *p*-value of a site is computed from the
  the match score of the site with the
  position specific scoring matrix
  for the motif.
  The *p*-value gives the probability of a random string
  (generated from the background letter frequencies) having the
  same match score or higher.
  (This is referred to as the **position *p*-value**
  by the MAST algorithm.)
- #### Block Diagrams of Motif Occurrences

  The occurrences of the motif in the training set sequences are
  shown with MAST-style block diagrams. One diagram is printed for each
  sequence showing all the occurrences of the motif in that sequence.
  The sequences are sorted by the **lowest** *p*-value among
  all occurrences of the motif in a given sequence.
  (The *p*-value of an occurrence is the probability of a single
  random subsequence the length of the motif,
  generated according to the 0-order background model, having a score
  at least as high as the score of the occurrence.)
  When the DNA strand is specified, '+' means the motif appears from
  left to right on the sequence, and '-' means the motif appears
  from right to left on the complementary strand.
  A sequence position scale is shown at the end of each table of block
  diagrams.
  Very long sequences are shown with thick lines connecting the
  motifs and are **not** drawn to scale.
- #### Motif in BLOCKS format or FASTA format>

  For use with
  BLOCKS tools,
  MEME prints the occurrences of the motif in BLOCKS format.

  You can convert these blocks to
  PSSMs (position-specific scoring matrices),
  LOGOS (color representations of the motifs),
  phylogeny trees and search them against a database of other
  blocks by pasting everything from the "BL" line to the "//"
  line (inclusive) into the
  Multiple Alignment Processor.

  If you include the
  **-print\_fasta**
  switch on the command line, MEME prints
  the motif sites in FASTA format instead of BLOCKS format.
- #### Position-Specific Scoring Matrix

  The position-specific scoring matrix corresponding to the motif
  is printed for use by database search programs such as MAST.
  This matrix is a log-odds matrix calculated by taking 100 times
  the log (base 2) of the ratio *p/f* at each position in
  the motif where *p* is the probability of a particular letter
  at that position in the motif, and *f* is the background
  frequency of the letter (given in the
  command line summary
  section.)
  This is the same matrix that is used above in computing the
  *p*-values of the occurrences of the motif in the
  Occurrences of the Motif
  and
  Block Diagrams of Motif Occurrences
  sections.
  The scoring matrix is printed "sideways"--columns
  correspond to the letters in the alphabet (in the same order as
  shown in the simplified motif) and rows corresponding to the
  positions of the motif, position one first.
  The scoring matrix is preceded by a line starting with
  "log-odds matrix:" and containing the length of the alphabet, width
  of the motif, number of characters in the training set, the scoring
  threshold (obsolete) and the motif *E*-value.

  **Note:** The probability *p* used to compute the PSSM
  is *not* exactly the same as the corresponding value in the
  Position Specific Probability Matrix (PSPM).
  The values of *p* used to compute the PSSM take
  into account the motif prior, whereas the values in the PSPM are just
  the *observed* frequencies of letters in the motif sites.
- #### Position-Specific Probability Matrix

  The motif itself is a position-specific probability matrix giving,
  for each position in the pattern, the observed frequency
  ("probability") of each possible letter.
  The probability matrix is printed "sideways"--columns
  correspond to the letters in the alphabet (in the same order as
  shown in the simplified motif) and rows corresponding to the
  positions of the motif, position one first.
  The motif is preceded by a line starting with
  "letter-probability matrix:" and containing the length of the
  alphabet, width of the motif, number of occurrences of the motif,
  and the *E*-value of the motif.

  **Note:** Earlier versions
  of MEME gave the posterior probabilities--the probability after
  applying a prior on letter frequencies--rather than the observed
  frequencies.
  These versions of MEME also gave the number of *possible*
  positions for the motif rather than the actual number of occurrences.
  The output from these earlier versions of MEME can be distinguished
  by "n=" rather than "nsites=" in the line preceding the matrix.
- #### Regular Expression

  This is a regular expression (RE) describing the motif. In each column,
  all letters with observed frequencies greater than 0.2 are shown;
  less-frequent letters are not included in the RE.
  MEME regular expressions are interpreted as follows:
  single letters match that letter; groups of letters in square brackets
  match any of the letters in the group.
  Regular expressions can be used for searching for the motif in
  sequences (using, for example,
  PatMatch
  ) but the search accuracy will usually be better with the PSSM (using,
  for example
  MAST.)
- #### Motif Summary Tiling

  The motif summary tiling is done using the same algorithm as used by
  MAST.
  The motif occurrences shown in the motif summary
  **may not be exactly the same as those reported in each motif section**
  because only motifs with a position *p*-value of 0.0001 that
  don't overlap other, more significant motif occurrences are shown.
  The format of the machine readable motif-summary is:

  ```
          [sequence_name combined_p-value number_of_motif_occurrences [motif_number start_of_motif position_p-value]+]+
  ```

  See the documentation for
  MAST output
  for the definition of position and
  combined *p*-values.
